# The Roulette Wheel: An Aid to Informed Decision Making

**DOI:** 10.1371/journal.pmed.0030137

**Published:** 2006-06-13

**Authors:** Jerome R Hoffman, Michael S Wilkes, Frank C Day, Douglas S Bell, Jerilyn K Higa

## Abstract

The authors outline a new visual tool that can help patients assess the benefits and risks of different treatments.

The old model of physician as authority figure dispensing advice and recommendations in the expectation that these will be followed precisely by patients has, for the most part, fallen out of favor. Such an image of physician as benevolent (or beneficent) dictator has been challenged by concern for patient autonomy, and shared decision making (SDM) has largely been adopted as an ideal way for physicians and patients to join together whenever there are decisions that need to be made about management of health care issues. SDM, in its best form, requires an informed and skillful physician, who is aware of the best available scientific evidence from clinical research, and is expert at communicating with patients in a meaningful, comprehensive, and non-distorted manner [1,2].

Such a physician must also be familiar with a series of concepts related to risk, rather than merely a set of facts. In the case of a choice about use of a screening test, an ideal physician would understand the generic circumstances in which screening is likely to be helpful or harmful, available data about the specific screening test, and subsequent possible interventions should it prove to be positive. This physician must also understand the problems patients typically have when trying to comprehend medical, and numerical, information, as well as the potential biases introduced during most doctor–patient communication [3–6]. Finally, he or she must attempt to understand the personal values and preferences of the patient, and to ensure that these are incorporated into the final decision (all the while being aware of his or her own values and preferences, so as to contribute as a trusted advisor, rather than as a mere robotic provider of information).

There are, in actual practice, many other limits to what is euphemistically called “informed” decision making. For many clinical questions, available evidence is insufficient or possibly even misleading. Most physicians are not ideally informed about the details of many clinical questions, lack sophistication in interpreting clinical research, are poorly conversant with the categorization of test characteristics and the application of Bayesian thinking, and may be ill-prepared to consider ethical problems that arise from many decisions. Patients, likewise, are frequently poorly informed, at best, or even misinformed because they have heard biased information from interested parties, such as direct-to-consumer advertising or advocacy groups [7]. Many patients do not have sufficient educational background to understand easily and quickly the complex issues that are often involved in medical decisions—nor in many cases are they even aware that such decisions can be complex and frequently do not reflect a single “right answer.” To make matters worse, there is rarely adequate time to spend on the many aspects of such a delicate consultation.

Although SDM is now identified as a desirable goal in many medical circles, a simpler process of informed consent may be appropriate, or even ideal, in “high-consensus” scenarios, such as when a vast majority of reasonable and educated physicians and patients would be expected to agree that the likelihood of benefit outweighs the risk of harm with a particular management strategy, compared with any other strategy. But even efforts to “inform” patients are frequently not observed or are honored in the breach. In many instances, a physician will seek to obtain “informed consent” simply by presenting a scenario along the lines of “here's what we have to do, and why” [6]. This may or may not be accompanied by a brief mention of the possible risks of the proposed strategy, and in rare cases, even by a very brief mention of potential alternatives—although this last element would typically be followed by a description of why such alternatives are not really reasonable.

Even when SDM seems ideal—as with a “low-consensus” scenario, a non-emergent situation, a patient capable of understanding a reasonable amount of information, and particularly an instance where an individual's personal values would be expected to weigh strongly—the barriers to such a process are obviously profound. Perhaps the greatest impediment involves the difficulty of providing information in a way that patients can understand, and which does not bias their response. Lack of time is not the only hurdle in such a process; there are many well-described ways (labeling, framing effect, etc.) in which standard communication can distort the manner in which information is perceived [3–6]. This is exacerbated by all sorts of problems related to most people's (doctors' as well as patients') difficulties in making sense of numbers. Most people also have difficulty understanding concepts of probability and risk, particularly as they relate to outcomes that are ultimately binary in any given individual.

Finally, attempts to “balance” possible harms and benefits associated with various approaches basically involve giving all potential outcomes—both good and bad—some artificial weight, perhaps calculated in light of the exploration of a separate set of “patient values,” and then attempting to determine which possible alternative strategy is “preferred” by comparing the calculated pluses and minuses [8–10]. Such techniques are commonly employed in clinical research, where “quality-adjusted life years” and the “standard gamble,” for example, each rely on our ability to “measure” various harms and benefits against some outside “gold standard.” This is fraught with hazard, of course, because asking individuals to estimate something on the order of “how many years of being alive, but in chronic pain, would you give up to attain one extra year of ‘perfect health’” is confounded not only by individual variation, the degree of chronic pain, the quality of life if health is “perfect,” and a person's baseline state of health or disability, but also by the impossibility, for most of us, of truly understanding the terms of such a question.

Many of us also have trouble understanding numbers, and what they actually imply, particularly in the context of risk and probability. It is hard for anyone to comprehend the difference between a 7% chance and an 8% chance—is there a meaningful difference?—and this is exacerbated when we try to deal in more extreme probabilities, such as “3 in 10,000.” There is no “unbiased” way to present “a 4% chance of death” as opposed to “a 96% chance of survival,” even though we are likely to hear these two versions of this one piece of information very differently. Some recent efforts to stress absolute, rather than relative, changes in risk, are helpful, and the use of “number needed to treat,” for both benefit and harm, can allow us to put all the effects of a given strategy into some perspective. But even this is far from optimal, since it requires us to try to balance equations like “one extra patient in 250 will not have a heart attack, while one extra patient in 12 will stop the medication due to adverse effects, and one extra in 1,000 will develop kidney failure.” Furthermore, such “explanations” only help to understand *population* risks, and don't help an individual patient choose between management strategies that each have a unique set of potential benefits and harms.

We therefore felt that it would be useful to create a tool whereby patients could make a choice between different strategies, based on a simple visualization of the probable outcomes associated with each of them. There is some evidence that visual tools, of various sorts, can enhance aspects of communication about risk [11]; in addition, we thought it important that the tool in question not require efforts to weigh, indirectly, various harms and benefits, or to understand concepts of probability and risk, but to rely simply on a *direct* appreciation of the differences in likely outcomes. Thus we created the “roulette wheel” model of probabilities, which provides viewers a simple visual tool upon which to base choices. (Of course the utility of this tool may be diminished among patients with visual impairment, such as color-blindness, or perhaps cataracts.)

For demonstration purposes, for the rest of this discussion, we present what we feel are reasonable estimates, on the basis of available evidence, about the effects of first-time prostate-specific antigen (PSA) screening for prostate cancer in an asymptomatic 65-year-old man, using the visual tools described below. Our numbers are based on the following assumptions, taken as much as possible from the medical literature: the prevalence of prostate cancer that would be fatal within 10 years, if untreated, is 2%; the prevalence of prostate cancer that would not be fatal within 10 years is 20%; the sensitivity of a PSA screening test is 100% for the fatal form of cancer and 30% for the nonfatal form; treatment results in a 50% reduction in the chances of dying from an otherwise fatal cancer; and the chances of suffering erectile dysfunction or incontinence because of treatment is 58% [12–14]. For this paper, we chose to assume that treatment of a PSA-detected cancer has benefit, although this is currently uncertain, because otherwise (no benefit, real harm) the choice not to offer or undergo screening would be straightforward. We have not considered such issues as cost, patient anxiety, problems with false negative tests, or adverse effects of biopsy in this model. All the tools described in this paper can be adapted to represent any current clinical question.

In this paper, all numbers are presented as point-estimates. Modern computer technology, however, allows these tools, when displayed on an internet Web site (http://edoctoring.ncl.ac.uk/System_Check/psa_detect_html; click on “Roulette Wheels”) or computer hard disk, to respond to changes in any and all of the assumptions being made, about such things as test characteristics, treatments, and outcomes, allowing for an infinite number of sensitivity analyses. Thus, both physicians and patients can work with not only a base set of probabilities derived from “best current evidence,” but also with the new sets of probable outcomes that would pertain if they wished to imagine the “best” and “worst” scenarios, using confidence intervals from clinical research, or to see what would be relevant for an individual whose baseline risk was higher or lower than that of an average member of a large cohort, or if new and better evidence is published, or if new and better tests, or treatments, become available.

## The Visual Tools

The most basic precursor of the roulette wheel is a simple pie chart (Figure 1), in which best estimates of various outcomes associated with different possible strategies can be depicted. When pie charts for two distinct strategies are shown side by side, the increase or decrease in likelihood of any particular outcome is easily and immediately visible to the observer. But presenting information in terms of two neighboring pie charts is suboptimal, because it fails to engage the viewer in terms of the relative *risk* associated with each of different options. Asking a patient to place himself in one of two populations, based on their respective population-based outcomes, is unlikely to simplify patient choice. The identical information, presented on dartboards (Figure 2), should be far more understandable to most patients, because most people would have little trouble with the concept that the result of throwing a dart would be generally proportional to the areas on a given board that provide a good reward (as in a high score) or a bad one (or even a punishment). To make the concept more familiar, and to increase the understanding that there is a risk involved with any “throw,” we spread the possible outcomes throughout the 360 degrees of the chart, in proportion to their likelihood of occurring (based on the numbers derived as per above), for the strategy in question. (We understand that as the risks of different outcomes are randomly distributed around the dartboard, their relative areas may not be as obvious as they would be if grouped together; on the other hand, we feel this arrangement is more visually understandable, particularly in terms of expressing the likelihood of “risk” of any given outcome.)

**Figure 1:**
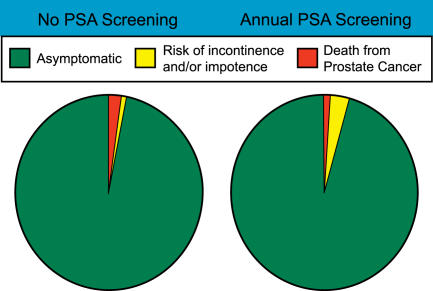
Pie Charts of Relative Risk for an Average 65-Year-Old Man, whether or Not He Opts for PSA Screening for Prostate Cancer Based on the best information available as of November 2005, the accuracy of which should be periodically revisited.

**Figure 2:**
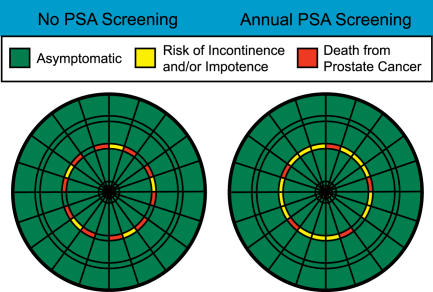
Dartboards of Relative Risks for an Average 65-Year-Old Man, whether or Not He Opts for PSA Screening for Prostate Cancer Based on the best information available as of November 2005, the accuracy of which should be periodically revisited.

Using this model, a patient would be asked to choose from among two (or more) competing dartboards the one at which he would be most comfortable aiming. In each case, in the example, most of the boards are covered with green, representing healthy survival, but each has a few dangerous areas that represent either morbidity (incontinence or impotence, in yellow) or mortality (in red). The “do a PSA” dartboard in Figure 2 (right side) has a slightly smaller red area, representing a slightly decreased likelihood of death, but the yellow area, representing impotence or incontinence, is substantially enlarged.

This dartboard model allows a patient (or physician) to respond directly to the choice offered by two different possible sets of risk, without having to rely on knowledge of numbers or percents, or having to assign artificial weights to various possible outcomes. The decision a patient makes in choosing at which dartboard he would prefer to throw his darts reflects his personal value system, in that he will respond to the relative degree to which the landing areas are filled with what he believes to be desirable or undesirable outcomes. Most patients will intuitively understand that it is preferable to throw at a board where most of the outcomes are positive, and worse to throw at one with larger portions covered with negative outcomes, particularly as those outcomes become more and more undesirable (with paralysis being worse than itching, for example).

Take for example a disease with a 40% mortality, which can be halved by a pharmacologic treatment, and that in turn causes minor side effects in 10% of patients (Figure 3). Anyone looking at the probable outcome associated with the two competing management options (use or do not use the treatment) can easily see that the total area of potential bad outcomes is diminished with treatment, and that even the “bad” outcomes associated with treatment are less fearsome than for no treatment. Similarly, even if 22% of patients develop a minor side effect with treatment (Figure 4), such that the overall green area is less with treatment, most patients will not need to make a conscious or explicit assignment of weights to “death” versus “minor side effects” to know that treatment is a more desirable choice.

**Figure 3:**
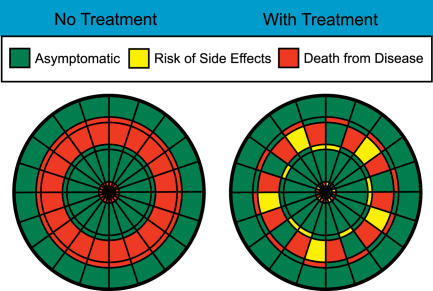
Dartboards of Relative Risks Associated with a Hypothetical Treatment

**Figure 4:**
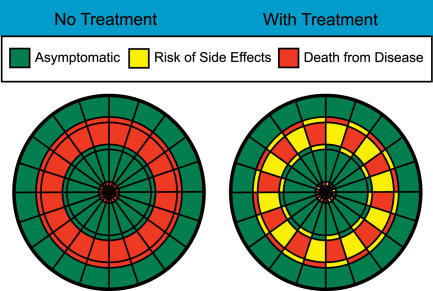
Dartboards of Relative Risks Associated with a Hypothetical Treatment (as in Figure 3) but with the Incidence of Side Effects Doubled

The situation is more complex, obviously, when the effects of two strategies are less straightforward, as in the case of prostate screening (Figure 2). In this case, it is perfectly feasible that one reasonable patient, noticing that (based on the assumptions we have made for the purposes of this paper) death is slightly less likely with screening, might choose to throw at the screening dartboard, even though the overall possibility of any bad outcome is increased. Another rational individual, on the other hand, might choose the no-screening dartboard, responding to the very small decrease in area covered by “death” and the much larger increase in area covered with significant morbidity.

For the physician, the advantages of allowing a patient to choose, based on use of such a direct visual tool, should be manifest. Particularly as choices become more and more complex, we cannot know on what basis any individual patient would go about choosing between competing options, and we can only delude ourselves by attempting to model the unknowable. It must be better to let an individual ruminate and come to his own conclusion, based on his own personal preferences, while attempting to assure that he has sufficient information as to the competing choices, and sufficient knowledge to weigh the relative advantages and disadvantages presented on each board.

There is, however, a problem with the dartboard metaphor, in that it makes it more difficult for a patient to understand that his own outcome, from among the range of possibilities represented, will be due to chance because darts is a game of skill, not chance. Thus it is possible that a patient who chooses to aim at a particular dartboard precisely because it, among available options, has the smallest area marked “impotence,” may nevertheless become impotent. Many patients would undoubtedly assume that they could impact the possibility of any given outcome because of their “skill” at aiming their own particular dart at the target area. Many patients already believe in their own ability to “beat the odds,” and this type of magical thinking would likely be accentuated by the notion of a game of skill, such as darts.

For that reason, we believe that the best version of this tool changes the terminology slightly so that the dartboard becomes a roulette wheel (Figure 5). For our interactive version, we have designed the tool to perform in the manner of an actual roulette wheel, so that for any given “spin” a ball turns around the board, and the spot at which the ball ultimately lands is entirely random. Most patients understand that there is no way to influence the final outcome by any one spin of such a wheel, although physicians would of course need to reinforce the message that by choosing to take their spin at any given roulette wheel, patients could have any of the outcomes seen on the wheel, but that the likelihood of any given outcome would be directly proportional to the area on the wheel taken up by the outcome in question. Patients could therefore look at two side-by-side roulette wheels and determine under which circumstances they would most like to spin. Once again, some patients might choose a wheel that maximizes the possibility of a good outcome, even if it has a slightly higher proportion of surface with some terribly bad outcome such as death, while others might choose to spin at a wheel where there were many less-than-perfect outcomes but the chance of death was smaller.

**Figure 5:**
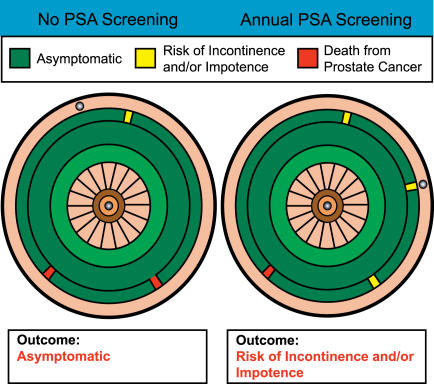
Roulette Wheels of Relative Risks for an Average 65-Year-Old Man, whether or Not He Opts for PSA Screening for Prostate Cancer Based on the best information available as of November 2005, the accuracy of which should be periodically revisited.

It is possible that for some patients magical thinking could be applied even to the roulette wheel, such that they would believe that the outcome they obtain on a sample spin (or series of spins) in a physician's office would predict what will actually happen to them should they choose to follow such a strategy “in real life.” It is also possible that other patients will object to using the roulette wheel because of religious (or other) objections to “gambling.” For both these reasons, it will be important for physicians to emphasize that the tool is designed only to demonstrate the likely potential hazards of alternate strategies, rather than to determine what will occur, for any individual patient, once a decision is made. Physicians may also choose to point out that, unlike the decision to go to a casino or racetrack, for example, no one can *avoid* making decisions about how to approach healthcare choices, or dealing with the potential risks and consequences associated with any given approach. Thus, using a tool like this has nothing to do with choosing to gamble with one's health, but rather has everything to do with understanding better just what risks are involved when any of several potential strategies (including inaction) is adopted.

We believe that the use of a tool like this, which would also allow for an infinite number of sensitivity analyses, and updating as new information became available, would greatly help patients understand the choices they have to make without requiring them to resort to some outside surrogate against which they would have to interpret these choices. It would also allow them to make decisions without having to understand concepts like “3% chance of death” or “97% chance of survival” or “25% decrease in chance of death” or “2% absolute chance of decrease in likelihood of death.”

Previous evaluations about the presentation of risk with visual tools are generally supportive of the use of visual tools for presenting risk [11,15], but this is not uniform, and there is also some concern that bias can result according to where on the chart (top or bottom) particular types of outcomes are placed [16]. We believe the dartboard is likely to diminish this concern, with risk information spread throughout the surface, and that the active roulette wheel is even less subject to this type of bias, as various outcomes end up on different parts of the surface in random, but proportionate, fashion following each spin.

Of course, use of the roulette wheel will not by itself solve all of the problems involved in SDM. It is still only as good as both the quality and precision of the available evidence, and it requires physicians who are able to interpret accurately the meaning of such evidence. It requires that patients understand that all outcomes are possible, including those that are of low likelihood, even though risk does occur in general proportion to the space on the wheel. Finally, although changes can be made to any of the parameters used to calculate the size of various outcomes on the wheel, it is hard to demonstrate multiple concomitant outcomes, or to visualize all the possible variations that are reasonable based on the best evidence. An example of this might be as follows: when a patient chooses to have a PSA, this might lead to increased worry while he waits for the result. This can easily be represented graphically on the wheel by adding another outcome category. On the other hand, if the test returns with a negative result, this same patient might worry less, over a subsequent period of time, than a patient who chose not to have the PSA in the first place. It would be very difficult if not impossible to model all possible responses in such a manner, especially without resorting to efforts to assign weights to the “degree of worry” that each approach might entail—precisely the type of activity the roulette wheel is designed to avoid. Nevertheless, we believe that major possible outcomes, both in terms of harm and benefit, can be visually represented using this device and, furthermore, that this can be done within a range of possibilities that are reasonable based on best available information.

It goes without saying that the roulette wheel only addresses that aspect of SDM that has to do with the ability of patients to understand information. It cannot solve problems related to the absence of reliable information, misinterpretation of information, or complex interactions of outcomes. Nor can it, nor should it, remove the physician from the equation, or take the place of doctor–patient discussions, which represent a substantial part of the contribution that physicians actually make when helping patients make important decisions. It can, however, help alleviate some of the substantial time pressures faced by physicians, by allowing patients to grapple with some of their own preferences and values independently, once appropriate data has been entered into the tool, prior to meeting with their physician, at which time questions can be raised, and areas of uncertainty addressed, without a need for the exhaustive review of risks and benefits associated with various choices that must underpin current attempts at SDM.

In this paper we can only demonstrate a static version of the roulette wheel as a tool for understanding risk, but it is worth reemphasizing that an interactive version (http://edoctoring.ncl.ac.uk/System_Check/psa_detect_html; click on “Roulette Wheels”) carries with it the further advantage of allowing physicians and patients to reconsider their opinions in light of uncertainty about the evidence, as well as once new evidence, or advances in diagnosis or treatment become available. Knowing, for example, that our point estimates are far from certain, a user of the tool could program it, quite easily, to show the relative outcomes presented by the “screen” and “no screen” options for a range of currently plausible assumptions. Some changed assumptions will favor one strategy, while others will do the opposite; seeing the range of possibilities, even for extremely favorable or extremely unfavorable assumptions, might be useful in helping some individuals refine their decision.

Even more importantly, this tool allows for rational adjustments based on new information, as it becomes available. The development of a more accurate screening test, or a more effective therapy for cancer, or a new intervention with a better adverse effect profile, would each be immediately and easily amenable to revisions in the paired roulette wheels, and viewers could reconsider the options in light of the new information. This would be hardly necessary in the face of dramatic advances, such as a new test that identifies only those cancers likely to become clinically relevant, or a new surgical technique that has no morbidity. But it would be of great value when considering new interventions that produce “advances” that are far more subtle: the roulette wheel could be a great help in understanding the impact of a new screening test shown to identify far fewer of the types of cancer that generally remain indolent for many years, but at the cost of lesser sensitivity for clinically important disease, or of a new therapy that produces far less impotence, but only decreases five-year, but not ten-year, mortality. (For offices able to use only the static version of the tool, it does contain a “warning” that it is based on the best information available as of a given date, the accuracy of which should be periodically revisited.)

For physicians and their patients to make informed decisions, they must not only have access to the best available information, but also must also understand what it means, as well as the possible consequences of the choices open to them. Like others, we believe that visual tools can be an extremely useful part of this process, particularly when they allow us to bypass the need to understand numerical probabilities, or to “translate” such numbers into artificial and often equally obscure surrogate “utilities” [11,17–19]. The programmable “roulette wheel” tool described in this paper should allow patients and physicians to incorporate directly their own values about competing benefits and competing harms, and thus lead to choices that are most consistent with their aspirations and fears, as well as with the best available evidence, even as it changes over time.

## Abbreviations

PSA: prostate-specific antigen
SDM: shared decision making
